# Anxious Imagery in Children With and Without Autism Spectrum Disorder: An Investigation into Occurrence, Content, Features and Implications for Therapy

**DOI:** 10.1007/s10803-016-2840-3

**Published:** 2016-06-21

**Authors:** Ann Ozsivadjian, Matthew J. Hollocks, Jess Southcott, Michael Absoud, Emily Holmes

**Affiliations:** 1 Children’s Complex Neurodevelopmental Disability Service, Children’s Neurosciences Centre Newcomen at St Thomas’, South Wing, St Thomas’ Hospital, Guy’s and St Thomas’ NHS Foundation Trust, Westminster Bridge Road, London, SE1 7EH UK; 2 Department of Clinical Neurosciences, University of Cambridge, Cambridge, UK; 3 MRC Cognition and Brain Sciences Unit, Cambridge, UK

**Keywords:** Autism spectrum disorder (ASD), Anxiety, Mental imagery

## Abstract

Mental imagery has been implicated in anxiety disorders in adults, but has not been investigated in child and adolescent populations. Anxiety is highly prevalent in autism spectrum disorder (ASD), and as people with ASD are often thought of as ‘visual thinkers’, the potential role of distressing imagery in children with ASD merits exploration. Participants aged 8–16 years were grouped as follows: ASD/high anxiety, ASD/low anxiety, non-ASD/high anxiety and non-ASD/low anxiety. Imagery and associated features were assessed using an interview. Group differences were found in number and frequency of images experienced. There were few differences between the groups in the characteristics of the spontaneous images, which included emotional valence, vividness, controllability and realism. Implications for treatment are discussed.

## Introduction

Emotional mental imagery is present in range of anxiety disorders (Hirsch and Holmes 2007; Holmes and Mathews 2010). Images have a more powerful impact on emotion than verbal thoughts, and are perceived as more realistic (Mathews et al. 2013). In adulthood, clinical groups compared to controls show higher frequency of intrusive images and greater negative emotionality associated with those images (Moulds and Holmes 2011). Few studies to date have investigated mental imagery in emotional disorders during childhood and adolescence. Studies that have investigated anxious imagery in young people have focused on manipulating a given image, rather than investigating the presence of existing, spontaneous imagery (Vassilopoulos et al. 2012). In typically developing children, imagery is known to play a role in cognitive and social development, such as the role of imaginary friends in perspective taking (Bouldin and Pratt 1999; Burnett Heyes et al. 2013). Given that anxiety disorders such as social anxiety and generalised anxiety disorder are common in childhood, and that anxiety disorders in adulthood often begin in childhood, this is an important area for exploration (Cartwright-Hatton 2013; Kessler et al. 2005).

In young people with ASD, prevalence studies have established that anxiety disorders occur in approximately 40 % of cases (van Steensel et al. 2011), which is significantly elevated compared to the general paediatric population rate of 2–3 % (Costello et al. 2003). It is now widely recognised that co-occurring anxiety disorders in ASD often cause more distress and can have a greater impact on individuals than the core symptoms of ASD themselves (Ozsivadjian et al. 2012). This underscores the need to better understand the psychological mechanisms that could be maintaining such anxiety.

Current research suggests that while there are many differences in the presentation of anxiety in the ASD population compared to typically developing children, there are also many overlaps and similarities. For example, children with ASD often experience typical specific phobias such as animal fears, but also frequently experience anxiety around change of routine, and unusual fears such as a fear of graffiti or sensory-driven fears (Kerns et al. 2014). As yet, however little is known about overlaps and similarities in pathways to anxiety. Given that people with ASD are often thought of as being ‘visual thinkers’ in that they may have a natural bias to process information visually rather than verbally (Kunda and Goel 2011), the enhanced use or presence of mental imagery may be one plausible aetiological factor in the development or maintenance of anxiety in ASD. It is therefore important to ascertain whether children with ASD might have excesses in imagery, and if so, whether these are associated with anxiety.

Children with ASD are also likely to experience more adverse life events such as bullying, have poorer autobiographical memory and more difficulty with emotion regulation than children without ASD, all of which may contribute to post-traumatic-type symptomatology including intrusive memories which are image-based (Kerns et al. 2015; Goddard et al. 2014; Mazefsky et al. 2013). Exposure to negative life events in adolescents (with no history of mental health problems) is strongly related to intrusive memories, the number and quality of which are related to more symptoms of depression (Meiser-Stedman et al. 2012). However, critical questions remain about the presence of emotional mental imagery in children both with and without ASD, and its predicted relationship to anxiety in both groups.

Therefore, for the first time, this study seeks to investigate whether children with and without ASD, and with high and low anxiety experience spontaneous anxious imagery, and if so, to what extent. Using a semi-structured interview, participants were asked about images in three scenarios; a relaxed scenario, a suggested anxious situation and about spontaneous images, if any were reported. The primary aim of this study was to investigate firstly, what proportion of children in each of the four groups experience anxious imagery, and whether there are differences between groups in terms of number of images and frequency with which they are experienced. Secondly, we sought to investigate whether children with ASD experience anxious imagery differently compared to children without ASD by investigating between-group differences in the associated characteristics of anxious imagery, including negative emotional valence, controllability, realism, vividness and cognitions associated with the image.

## Methods

### Participants

Forty-three children with ASD and 35 non-ASD children with a Full Scale IQ ≥ 70 were included in this study. Of the 43 ASD participants, 35 were medication free, 5 were on risperidone, 2 on a selective serotonin reuptake inhibitor (SSRI) and 1 on methylphenidate for management of behaviour that challenges which is representative of the ASD population (Frazier et al. 2011). Thirty-three of the ASD sample were recruited via a Complex Neurodisability clinic at a London Hospital, and 10 were recruited with the assistance of a specialist schools for children with ASD. The children recruited via the clinic all had diagnoses made using the Autism Diagnostic Interview-Revised and Autism Diagnostic Observation Schedule (2nd edition); those in the specialist school were not re-assessed via ADI-R/ADOS-2 as confirmed diagnosis was a requirement of school admission (Lord et al. 2000; Lord et al. 1994). However in both groups, for parity, ASD status was screened for using the Social Communication Questionnaire (SCQ) using the recommended cut-off of ≥15 (Berument et al. 1999). The ASD group was then divided into high anxiety (n = 29) and low anxiety (n = 17) using the SCAS-P recommended cut-off of 24 (Rodgers et al. 2012).

A group of non-ASD children with high-anxiety (n = 17) were recruited via other services across the Neurosciences centre which included the sleep service, tic disorders service, the feeding clinic, and children’s psychological medicine. Children were referred to the study as they were identified within their services for treatment with anxiety as a primary target, post initial assessment but prior to treatment. Absence of ASD was confirmed using the SCQ and the degree of anxiety (high or low) was confirmed using parent Spence anxiety questionnaires. Only children with SCQ scores below 15 and SCAS-P scores above the threshold of 24 were considered for this group. A further 18 anxiety-free non-ASD children were also recruited with the help of local schools and volunteers for a non-ASD low anxiety group. Only children with SCQ scores below 15 and SCAS-P scores below the threshold of 24 were considered for this group.

In total, there were four groups, ASD high anxiety (mean age 11.9 years; 23 male, 6 female); ASD low anxiety (mean age 13.1 years; 12 male, 2 female); non-ASD high anxiety (mean age 12.0 years; 8 male, 9 female) and non-ASD low anxiety (mean age 11.0 years; 11 male, 7 female). Only children with intellectual functioning above 70 were included, to ensure adequate understanding of the interview questions.

### Measures

The Spence Children’s Anxiety Scale, Parent and Child Versions (SCAS-P and SCAS-C) is a 44-item questionnaire widely used both clinically and in research to screen for anxiety disorders (Spence 1998). Results have been found to have good internal consistency (α = 0.60–0.92) and high test–retest reliability (α = 0.45–0.62). Although there is no formal clinical cut-off on the SCAS-P, a cut-off of 24 has been has previously been used as an indicator of clinical caseness (Rodgers et al. 2012) based on normative data (Nauta et al. 2004) and personal communication. The SCAS is one of the few measures of emotional functioning to have been validated on an ASD sample (Zainal et al. 2014), showing moderate to good convergent and discriminant validity. In the current study, internal consistency was good or acceptable for both the self- (ASD α = 0.91; Non-ASD α = 0.93) and parent-report (ASD, α = 0.75; Non-ASD α = 0.89) in both the ASD and non-ASD samples.

The Children’s Depression Inventory (CDI) parent and child version is a symptom-orientated questionnaire to screen for depression in children aged 7–17 years. Internal consistency is high (α = 0.86) (Kovacs 1992). In the current study, internal consistency was good or acceptable for both the self-report (ASD α = 0.93; Non-ASD α = 0.92) but poor for parent version in ASD group only (ASD, α = 0.58; Non-ASD α = 0.76). This measure was included for a descriptor variable only and not included in the analyses pertaining to the hypotheses.

The SCQ is a brief screening instrument useful when assessing social and communication skills in children with a suspected ASD. This measure is completed by a parent and takes approximately 10 min to complete. A cut-off score of 15+ is often used to identify children and adolescents who display characteristics of ASD (Chandler et al. 2007). The SCQ had good internal consistency when used in both the ASD and non-ASD samples (ASD α = 0.95; Non-ASD α = 0.93).

The Spontaneous Use of Imagery Scale (SUIS) provides a trait measure of the use of non-emotional imagery in everyday life (Reisberg et al. 2003). A series of descriptions is given, for example: “When I think about visiting a relative, I almost always have a clear mental picture of him or her”. Each description is rated on a 5-point scale, from 1 = never appropriate to 5 = always completely appropriate. A total score is calculated.

#### Imagery Interview

This is a structured clinician-administered instrument developed originally by Hackmann et al. (1998), and subsequently modified by Day et al. (2004) which assesses the content and qualities of mental images and associated verbal thoughts. In this version adapted for children, participants were asked a set of questions about images in three scenarios; (1) a relaxed, self-generated situation, (2) a suggested anxious situation (giving a presentation to the class, or in assembly to the whole school) and (3) a spontaneously generated anxious situation where images were experienced. The relaxed situation serves as both a control and practice condition and we therefore focused only on the suggested anxious imagery and spontaneous anxious imagery scenario for analysis.

For each scenario, participants were asked to imagine and describe the scenario, and asked the same set of questions regarding ratings of emotions associated with the image generated, on a 1–9 scale. These were collapsed into a single variable for total emotional valence associated with the image with a total score ranging from 0 to 63 (positive emotions were reverse coded). Participants also gave 1–9 ratings for vividness, controllability, how ‘real’ the image felt, and how upsetting the image was.

For the spontaneous imagery section, participants were first asked whether there was a situation that made them anxious in which they had images, or whether they had images that made them anxious. If no spontaneous anxious image was reported, the interview was terminated at this point. If an image was reported, they were then asked how frequently they experienced the image, followed by the questions about emotional valence and other associated factors. An additional series of questions regarding the meaning and impact of the image were asked, including ‘does the image make you want to do anything?’ and ‘Does the image say anything about you?’. We also asked whether the image was related to an actual event. Finally, participants were asked to estimate roughly how many other images they experience (1, 2–5, 6–10, >10).

### Measures of Cognition

#### Wechsler Abbreviated Scale of Intelligence (WASI)

The WASI was selected as a reliable but brief measure of intelligence; the two item version was administered. This form contains only the Vocabulary and Matrix Reasoning items but provides an estimate for full scale IQ (FSIQ) (Wechsler 1999).

### Procedure

The study was approved by the London and South East research ethics committee (Reference: 13/lo/0104). Participants were approached via their clinician to ascertain initial interest; information sheets were sent to participants by post or email in order to allow them to make an informed decision and contact the researchers independently with consent. Written consent was then obtained from parents and their child at the appointment, prior to commencing the procedure.

The order of administration was as follows: adapted imagery interview, followed by the cognitive tasks from the WASI, followed by questionnaire measures. The procedure was concluded by debriefing and a brief computer game to relax participants prior to departure. The procedure took approximately 1 h and interviews were conducted by either the principal author or one of two research students. Inter-rater reliability was not assessed as only quantitative data was analysed, however the students were fully trained and observed while administering the interview initially to ensure parity of administration. However, two transcripts were rated separately by the researchers regarding the content of the images; agreement as to whether images were typical/atypical was 100 %.

### Statistical Analysis

Groups were first compared on descriptive and baseline variables. Our inferential statistics are divided into three sets of analyses; (1) The sample was divided into four groups to compare group differences in the total amount of spontaneous anxious imagery and the frequency of those images in those with ASD (high and low anxiety) and the non-ASD groups (high and low anxiety), (2) then using both the spontaneous anxiety situation and the suggested anxious situation (giving a presentation to the class, or in assembly to the whole school), the four groups were compared on their response to images with regards to vividness, controllability, realism, and how upsetting their images were, (3) We then provide some examples of the content of anxious imagery in those with ASD compared to non-ASD participants. Then based on transcripts of the imagery interview participant’s images were classified as being related to either typical and atypical anxiety based on criteria suggested by Kerns et al. (2014), and group differences in these characteristics were quantified.

Parametric or non-parametric statistical tests (Kruskal–Wallis tests) were used for continuous distributions as appropriate given normality and χ^2^ or Fisher’s exact tests for nominal data. Ratings of vividness, controllability, how ‘real’ the image felt, and how upsetting the images were all positively skewed across the sample and therefore non-parametric Kruskal–Wallis tests were used. Levels of *p* < 0.05 were taken as significant. The primary analyses for analysis 1, consisted of two ordered logistic regression models to identify any group differences on the predicted variables of interest: frequency of spontaneous anxious imagery/number of anxious images experienced in the spontaneous imagery condition. Group was entered as a predictor, along with the covariates of age, FSIQ and SUIS total score, to predict (1) the frequency of spontaneous anxious imagery (ordinal variable coded: not at all; no. images monthly/yearly; no. images weekly; no. images hourly/daily) and (2) the total number of images (ordinal variable coded: none; 1–5; >5). The primary analysis for analysis 2 was a multiple linear regression predicting the total negative emotional valence to imagery. Effect sizes for all linear regression and ANOVA models are presented as ω^2^ (omega-squared), which is a suitable measure of effect size for analyses with a small sample size. This can be interpreted as follows: small effect = 0.01, medium effect = 0.06 and a large effect = 0.14. For analysis 3, Fisher’s exact tests were used to compare groups on rates of typical versus atypical anxieties and whether images consisted of actual experienced events versus make believe events.

## Results

### Descriptive Statistics

As expected there were significant group differences on the SCQ, SCAS-P/SCAS-C, but also on the CDI-P/CDI-C (see Table 1). There were no significant group differences in age or FSIQ, although gender did approach significance with more males than females in the ASD groups.

**Table 1:** Descriptive statistics mean (SD)

	ASD high anxiety (N = 29)	Non-ASD high anxiety (N = 17)	ASD low anxiety (N = 14)	Non-ASD low anxiety (N = 18)	
Age	11.9 (2.3)^a^	12.0 (2.1)^a^	13.1 (1.9)^a^	11.0 (2.2)^a^	F = 2.5, *p* = 0.07^‡^
FSIQ	102.9 (15.8)^a^	103.0 (15.7)^a^	112.1 (12.3)^a^	109.7 (11.1)^a^	F = 1.9, *p* = 0.12^‡^
Gender (%Male)	79 %	47 %	86 %	61 %	*χ* ^2^ (3) = 7.6, *p* = 0.05^†^
SCQ	23.0 (8.9)^a^	12.1 (9.7)^b^	21.8 (6.8)^a^	2.1 (1.9)^c^	F = 31.7, ***p*** **≤** **0.01** ^‡^
SCAS-P	45.3 (15.1)^a^	49.4 (23.4)^a^	15.0 (5.9)^b^	11.7 (6.1)^b^	F = 33.0, ***p*** **≤** **0.01** ^‡^
SCAS-C	39.8 (26.2)^a^	46.7 (21.1)^a^	30.1 (19.7)^a^	19.3 (12.0)^b^	F = 5.7, ***p*** **≤** **0.01** ^‡^
CDI-P	68.9 (11.6)^a^	72.1 (12.7)^b^	60.9 (12.1)^b^	46.3 (8.0)^c^	F = 19.8, ***p*** **≤** **0.01** ^‡^
CDI-C	64.3 (12.8)^a^	63.9 (13.8)^a^	54.3 (5.8)^b^	49.1 (7.7)^b^	F = 9.4, ***p*** **≤** **0.01** ^‡^
SUIS total Score	32.9 (8.4)^a^	31.1 (7.9)^a^	27.4 (5.3)^a^	29.3 (7.4)^a^	F = 1.8, *p* = 0.16^‡^

^†^Chi-squared test; ^‡^ One-way ANOVA

Significant group differences at *p* < 0.05 are presented in bold (a > b > c)

#### Are there Between-Group Differences in the Total Number and Frequency of Spontaneous Anxious Images?

The percentage of participants in each of the four groups reporting at least one image in the spontaneous anxious imagery scenario was 86 % of the ASD high anxiety group, 76 % of the non-ASD high anxiety group, 62 % of the ASD low anxiety group and 44 % of the non-ASD low anxiety group. There was a significant difference between groups [*χ* ^2^ (3) = 10.0, *p* = 0.02]. Within those who reported having at least one spontaneous anxious image, when controlling for age, FSIQ and SUIS total score, there were significant between-group differences in the total number of other anxious images reported [ordered logistic regression: *χ* ^2^ (6) = 28.6, *p* ≤ 0.01, see Table 2]. The ASD high anxiety group (β = 2.7; *p* ≤ 0.01), non-ASD high anxiety group (β = 1.8; *p* = 0.01) and ASD low anxiety group (β = 2.5; *p* ≤ 0.01) all had more other anxious imagery when compared to the non-ASD low anxiety group but there were no significant differences between the three groups. None of the covariates were significantly related to the number of anxious images reported.

**Table 2:** Ordered logistic and linear regression models investigating group differences in anxious imagery and emotional valence controlling for age, FSIQ and use of non-anxious imagery

	ASD high anxiety (N = 29)	Non-ASD high anxiety (N = 17)	ASD low anxiety (N = 14)	Non-ASD low anxiety (N = 18)	Statistical test	Group differences
Spontaneous anxious imagery condition						
Other images (% none/1–5/>5)	10/57/33	18/76/6	28/36/36	61/49/0	*χ* ^2^ (6) = 28.6, ***p*** **≤** **0.01** ^‡^	ASDanx = nASDanx = ASD > nASD
Image frequency (%none/monthly-yearly/weekly/hourly-daily)	17/17/21/45	17/17/23/45	29/29/21/21	50/20/10/20	*χ* ^2^ (6) = 15.2, *p* = 0.02 ‡	(ASDanx = nASDanx) > (ASD = nASD)
Negative emotional valence (1–63)	34.9 (10.1)	41.9 (14.4)	28.1 (9.3)	28.4 (11.8)	β = 1.9, *p* = 0.24^†^, ω^2^ = 0.03	ASDanx = nASDanx = ASD = nASD
Suggested anxious imagery condition						
Negative emotional valence (1–63)	30.0 (9.7)	29.6 (14.8)	28.4 (16.6)	18.8 (7.4)	β = 3.3, ***p*** **≤** **0.01** ^†^ ω^2^ = 0.06	ASDanx = nASDanx = ASD > nASD^ш^

^†^Linear regression; ^‡^ ordered logistic regression

Significant group differences at *p* < 0.01 are presented in bold

^ш^ASDanx = ASD high anxiety; nASDanx = non-ASD high anxiety; ASD = ASD low anxiety; nASD = non-ASD low anxiety

Ordered logistic regression analysis revealed significant group differences in image frequency [*χ* ^2^ (6) = 15.2, *p* = 0.02, Table 2]. Specifically, that the ASD high anxiety group (β = 1.6; *p* = 0.01) and the non-ASD high anxiety group (β = 1.7; *p* ≤ 0.01) reported experiencing anxious imagery significantly more frequently than both the low anxiety groups who did not differ from each other (β = 1.0; *p* = 0.16). The ASD high anxiety group and the non-ASD high anxiety group also did not differ from each other. The SUIS total score was a significant predictor of image frequency (β = 0.7; *p* = 0.02).

#### Are there Between-Group Differences in the Characteristics and Associated Emotional Valence of Either Spontaneous or Suggested Anxious Imagery?

In the spontaneous anxious imagery condition, there were significant between group differences in how upsetting the participants found the spontaneous imagery (Table 3), with post hoc comparisons indicating that the ASD high anxiety group and the non-ASD high anxiety group did not significantly differ (z = 0.4, *p* = 0.69), but that both high anxiety groups scored significantly higher than the ASD low anxiety and TD low anxiety groups who did not differ (z = 0.72, *p* = 0.47) from each other (Table 3). There were no significant differences in the vividness, controllability or the realism of images between the four groups (although vividness did approach significance) nor significant differences in the use of non-anxious imagery measured using the SUIS [F (3, 74) = 2.16, *p* = 0.10]. There was a difference between groups in negative emotional valence attached to the image (Table 3), but when controlling for age, FSIQ and SUIS total score there was no significant effect of group nor were any of the covariates significantly related to negative emotional valence (see Table 2).

**Table 3:** Group differences in associated features of suggested and spontaneous anxious imagery

	ASD high anxiety (N = 29)	Non-ASD high anxiety (N = 17)	ASD low anxiety (N = 14)	Non-ASD low anxiety (N = 18)	
Spontaneous anxious imagery condition					
Vividness (0–9)	7.1 (2.1)^a^	6.4 (2.9)^a^	4.8 (2.4)^a^	4.9 (3.1)^a^	*χ* ^2^ (3) = 6.9, *p* = 0.07^†^
Controllability (0–9)	3.6 (3.1)^a^	2.9 (2.7)^a^	5.4 (2.7)^a^	3.5 (2.7)^a^	*χ* ^2^ (3) = 4.9, *p* = 0.17^†^
Realism (0–9)	6.4 (2.7)^a^	5.9 (2.9)^a^	4.6 (2.9)^a^	6.5 (2.8)^a^	*χ* ^2^ (3) = 2.5, *p* = 0.47^†^
Upsetting (0–9)	6.8 (2.5)^a^	6.9 (2.9)^a^	4.4 (2.6)^b^	3.8 (2.9)^b^	*χ* ^2^ (3) = 12.6, ***p*** **≤** **0.01** ^†^
Negative Emotional valence (1–63)	34.9 (10.1)^a,b^	41.9 (14.4)^b^	28.1 (9.3)^a^	28.4 (11.8)^a^	F (3, 54) = 3.8, ***p*** **=** **0.02** ^‡,^ ω^2^ = 0.10
Suggest anxious imagery condition					
Vividness (0–9)	5.9 (2.8)^a^	5.7 (2.7)^a^	4.9 (2.4)^a^	5.6 (2.1)^a^	*χ* ^2^ (3) = 3.4, *p* = 0.49^†^
Controllability (0–9)	4.6 (2.9)^a^	3.4 (2.6)^a^	4.7 (3.3)^a^	5.2 (2.1)^a^	*χ* ^2^ (3) = 3.5, *p* = 0.32^†^
Realism (0–9)	5.8 (2.5)^a^	4.6 (3.1)^a^	4.7 (2.8)^a^	4.0 (2.5)^a^	*χ* ^2^ (3) = 5.6, *p* = 0.13^†^
Upsetting (0–9)	3.4 (2.3)^a^	4.0 (3.1)^a^	3.3 (2.4)^a^	1.8 (1.3)^a^	*χ* ^2^ (3) = 6.1, *p* = 0.10^†^
Negative emotional valence (1–63)	30.0 (9.7)^a^	29.6 (14.8)^a^	28.4 (16.6)^a^	18.8 (7.4)^b^	F (3, 69) = 3.7, *p* **≤** **0.01** ^‡,^ ω^2^ = 0.10

^†^Kruskal–Wallis test; ^‡ ^one-way ANOVA

Significant group differences at *p* < 0.05 are presented in bold (a > b)

In the suggested anxious imagery condition, there were no significant group differences between the vividness, controllability, how upsetting the images were or the realism of the imagery (see Table 3). However, there was a significant between group differences in negative emotional valence. When controlling for the covariates, regression analysis (Table 2) revealed that this significance remained. The ASD high anxiety (mean = 30.0, SD = 9.7; β = 10.9; *p* ≤ 0.01) and non-ASD high anxiety (mean = 29.5, SD = 14.9; β = 10.4; *p* = 0.02) and ASD-low anxiety (mean = 28.4, SD = 16.6), groups showed greater negative emotional valence than the non-ASD low anxiety (mean = 18.8, SD = 7.4). Effect sizes for parametric analyses are shown in Tables 2 and 3.

#### Group Differences in the Content of Anxious Imagery

Kerns et al. (2014) define typical and atypical anxiety in ASD as being consistent with diagnostic criteria, for example, a fear of negative social evaluation in social anxiety, or inconsistent with diagnostic criteria, for example, worry specifically related to changes in routine. Using these definitions, examination of the content of the images yielded the following impressions. Children with ASD, both with and without anxiety, were more likely to report atypical fears in their images (see Table 4) however overall these differences were not significant between groups. They were significantly more likely to report an imagined event or object such as an intruder or internet character, rather than an actual memory or event. These group differences were significant. Examples of the content of images and related cognitions in the spontaneous imagery scenario are reported in Table 5.

**Table 4:** Group differences in the content of anxious imagery

	ASD high anxiety (N = 29)	Non-ASD high anxiety (N = 17)	ASD low anxiety (N = 14)	Non-ASD low anxiety (N = 18)	Fisher’s exact test
Typical/atypical content (n)	12/10	3/7	9/5	6/3	*p* = 0.39
Actual event/imagined event	16/6	2/8	10/4	6/3	*p* = **0.035** ^*^

* Significant group differences at *p* ≤ 0.05

**Table 5:** Examples of images, and associated cognitions

Participant	Image (typical/non-typical)	What does the image say about you, the world	Does the image make you want to do anything?
ASD, high anxiety	Alone on a bench at school, smell of my arteries popping (both typical and non-typical)	Maybe your Asperger’s is why you’re such a joke. Humanity sucks	Walk up to people, slap them and say how dare you
ASD, high anxiety	Faces—of people I know from school, and another one added in—one is grinning, one is petrified (atypical)	Possibly I’m a bad person, meaning when I die I’ll go to hell	It makes me want to stay still and not talk to anybody and go off somewhere
ASD, high anxiety	Humpty Dumpty—looked so ugly when it cracked. The eyes was the worst thing. Took months to get that picture out of my head.	Nothing	No
ASD, low anxiety	Someone not believing me, I’m trying to talk, their face is all blurred (typical)	I’m always in the wrong place at the wrong time	Makes me want to find out the truth
TD, high anxiety	Spiders, long hairy legs, eyes (typical)	I’m scared of spiders	Scream and cry
TD, low anxiety	Arguing with a friend (typical)	I’m insecure about popularity	Tell a friend I trust

## Discussion

Our study aimed to explore for the first time, whether anxious imagery occurs in children with and without ASD. Our results, in line with existing research in adult populations with anxiety disorders, suggest that children with high anxiety both with and without ASD, and interestingly, also those with ASD and low anxiety, do indeed report significant levels of spontaneous, intrusive anxious imagery. Children with ASD and high anxiety were the most likely to report to experience at least one spontaneous image, followed by non-ASD high anxiety, then ASD low anxiety and finally non-ASD low anxiety who were least likely to report any spontaneous anxious imagery. Children with high anxiety with and without ASD, and also those with ASD and low anxiety report a higher number of images when compared to non-ASD low anxiety children. Taken together, these findings suggest that children with ASD may have a greater propensity towards experiencing anxious imagery when compared to their non-ASD counterparts, even if they are not anxious. In addition, children with high levels of anxiety regardless of having an ASD diagnoses experienced anxious imagery with more frequency than both low anxiety groups.

In terms of the characteristics and associated emotions of the spontaneous images, there were few group differences, other than how upsetting the participants found the image, with both high anxiety groups rating their image as more upsetting than the low anxiety groups. Negative emotional valence associated with the image was also greater in these groups although this was not significant when age, IQ and SUIS scores were taken into account. This finding may indicate that spontaneous anxious imagery features, are likely to be similar regardless as to whether children have ASD or not.

In our study, if a child did not report a spontaneous image, the interview was terminated, therefore no data was gathered on spontaneous images for a large number of the non-ASD non-anxious group. Consequently the suggested scenario which was completed by all participants potentially yields important additional data. The three clinical groups (ASD high-anxiety, non-ASD high anxiety and ASD-low anxiety) all rated the suggested image as more emotionally salient than did the non-ASD non-anxious group. However there were no group differences in any other characteristic of the image. This indicates that when a potentially distressing image is generated, those with ASD may find this more distressing than their non-ASD counterparts. Given that children with ASD are more likely to report anxious imagery than those without, we would cautiously suggest, that an increased propensity to experience some anxious imagery, which has a negative emotional impact, may be one possible pathway to anxiety in ASD.

Although non-systematic, a qualitative examination of the data suggests that young people with ASD are no more likely to report atypical images, but are more likely to report imagined images (as opposed to based on an actual memory) than their non-ASD counterparts. We found this result interesting, given that people with ASD typically display imagination deficits (Scott and Baron-Cohen 1996). This finding is preliminary and needs more systematic evaluation, but suggests that images may follow the model of both typical and atypical presentation of anxiety proposed in the Kerns et al. (2014) study, in that there are both overlaps and differences in the content of imagery between ASD and non-ASD populations. Systematic evaluation of the content of images in future studies, perhaps including relaxed images as well as anxious ones, might further elucidate information in this regard.

Surprisingly, there were also no differences between groups on the use of non-anxious imagery in everyday life as measured by the SUIS, suggesting that although people with ASD are thought of as visual thinkers, this may not necessarily be borne out under scrutiny, or perhaps simply that verbal thinking is more compromised, rather than visual thinking enhanced. However numbers in our study are not large enough to reach a firm conclusion in this regard, and this finding needs to be replicated in future studies. The SUIS was found to be related to the frequency of experiencing spontaneous images (in both ASD and non-ASD groups), indicating that a greater use of imagery in everyday life is related to higher frequency of experiencing anxious imagery. The SUIS may hence serve as a useful additional measure for children who are experiencing anxiety.

Although the study results are preliminary in nature and need replication, the findings of our parametric analyses indicate a medium effect size which is promising for future studies. In addition, this data will help to generate further hypotheses to be tested. For example, do the situations give rise to anxious images or do images create anxiety in such situations? Research by Vassilopoulos et al. (2012) and also Hirsch et al. (2003) in adults, suggests that manipulating negative self-imagery does play a causal role in the development of social anxiety. However our research extends beyond self-images in socially anxious situations to a range of spontaneously experienced images, some of which have little relation to the self, and that appear to be related to broader anxiety symptoms. Further investigation regarding the relationship (and potential pathways) from anxious imagery to anxiety is warranted. Indeed, investigating whether or not similar relationships between negative imagery and depression would also be warranted.

The greatly elevated rate of anxiety disorders in this population suggests that cognitive, physiological or other factors (e.g. environmental) associated with the ASD profile may predispose individuals with ASD to an anxiety disorder. Nonetheless, anxiety is not universal in this population, indicating that anxiety in ASD is not inevitable, therefore the identification of possible risk factors and pathways to anxiety is important. Currently, many of these risk factors are speculative, although a small body of research is emerging investigating cognitive and physiological pathways to anxiety in ASD, and promising avenues of research include intolerance of uncertainty (Boulter et al. 2014) and attentional bias (Hollocks et al. 2013). Anxious imagery may have either a mediating or causal role in the development of anxiety, for example, by the images themselves leading to fears, or by promoting maladaptive coping styles such as avoidance or suppression of certain images. Images could potentially lead to some of the idiosyncratic fears that are often seen in ASD, as we found that more young people with ASD reported imagined images, rather than actual memories, than those without. Images could also lead to fear via contextual conditioning. For example, one young man reported images with scary eyes that bothered him nightly. This could potentially lead to an aversion or preoccupation with eyes in everyday life. Alexithymia, which refers to a difficulty with emotion recognition or emotional processing and commonly associated with ASD (Bird and Cook 2013) might further exacerbate the impact of images, for example by eliciting maladaptive responses to anxious images.

### Limitations

An important limitation was that anxiety was not measured using a formal interview. Therefore children were grouped as anxious or non-anxious, as numbers were not large enough to establish sub-types of anxiety. However, in our view this reflects the reality of clinical practice; rarely do children present with single anxiety disorders that fit perfectly into a model. Although this limits how specific we can be about the type of imagery and the pathways to specific disorders, it does not diminish from the overall finding and recommendation that children with anxiety experience images that can and should be included in a treatment protocol. However, future research would benefit from inclusion of a standardised diagnostic interview.

Another important limitation was that the non-ASD, anxious group was recruited from clinics within a neurosciences centre. Unfortunately it was beyond the scope of this study to do a full screen or gather systematic data on co-occurring problems other than their primary referral problem. Future studies would benefit from recruitment from other sources such as a community-based anxiety clinic, with robust screening for co-morbidities.

### Clinical Implications

Several trials have shown that various CBT protocols for anxiety can be effective in children with ASD (Sukhodolsky et al. 2013). CBT for children traditionally tends to focus on verbal techniques for eliciting thoughts and modifying dysfunctional beliefs. Our research suggests that exploration of imagery is an important tool both for direct intervention, in using imagery restructuring techniques to modify unwanted, intrusive images, and also indirectly, for accessing verbal cognitions that otherwise might be difficult to access. For example, if a child states that the image means ‘I am useless’, they could be asked whether they often feel that way, and in what other situations. Hence, treatment which utilises brief imagery restructuring can be a powerful intervention in reducing both the presence and impact of distressing images (Holmes et al. 2007). However, whether there is an additional benefit of restructuring of specific images on reduction of overall anxiety needs investigation, as does whether imagery probing does in fact yield more in the way of dysfunctional beliefs than traditional verbal techniques.

The impact of anxiety and associated images was not specifically measured in this study, but is possible to mention anecdotally, as many of the participants in this study went on to receive treatment in the centre. For example, one child who reported anxious images of arriving at school late and being stared at, had been out of school for 6 months due to anxiety around school. Another child who reported images of the eyes from a cartoon character, had seen the character in question some 6 years previously but was still troubled nightly by the eyes. The red spot on the hand reported by one child resulted in obsessions around blood and decontamination rituals, and the child reporting an image of someone looking at them while reading a magazine, was experiencing thoughts about others bordering on paranoia, which was hindering him accessing public transport. Previous research by Hales et al. (2011) found a relationship between suicidal images and the desire to act on them in a sample of bipolar adults. Therefore, it is likely that the images themselves are a legitimate and worthy target for intervention, possibly as a first line treatment for children who are having difficulty accessing CBT due to, for example, verbal limitations.

## Conclusions

These results are novel in that anxious children with and without ASD experience anxious imagery, and that children with ASD may be more likely to experience images than those without. However there is little difference between the clinical groups in terms of characteristics of spontaneous images, although the content of these images suggested that children with ASD may experience more imagined images, rather than images based on actual events, than those without. Therefore this demonstrates both similarities and differences between children with and without ASD in the experience of anxious imagery. Given the evidence for a role in mental imagery in a range of emotional disorders, many of which frequently have onset in adolescence, a developmental understanding is important. The high prevalence of anxiety in ASD, and the challenges for them in accessing ‘talking therapies’, mean that this study also has implications for clinicians providing psychological support to children with ASD. Imagery restructuring can be a very powerful tool for dealing with both unwanted, intrusive imagery, as well as giving access to cognitions that may be challenging to reach via verbal techniques, especially in verbally-challenged populations, as children with ASD tend to be. Therefore an important clinical implication of this research is that the presence of anxious imagery should always be probed in treatment, and a trial investigating the efficacy of imagery-enhanced CBT in children and young people with anxiety is warranted.
